# Negatively Linking Connector Networks in Cognitive Control of Affective Pictures

**DOI:** 10.3389/fnins.2019.01069

**Published:** 2019-10-25

**Authors:** Wenhai Zhang, Jing Chen, Guofang Ren, Fanggui Tang, Qiang Liu, Hong Li

**Affiliations:** ^1^College of Education Science, The Big Data Centre for Cognitive Neuroscience and AI, Hengyang Normal University, Hengyang, China; ^2^Research Center of Brain and Cognitive Neuroscience, Liaoning Normal University, Dalian, China; ^3^Institute for Brain and Psychological Sciences, Sichuan Normal University, Chengdu, China; ^4^School of Education, Anyang Normal University, Anyang, China; ^5^College of Psychology and Sociology, Shenzhen University, Shenzhen, China

**Keywords:** affective pictures, cognitive control, connector hubs, graph theory, negative connections

## Abstract

Cognitive control of emotions depends on intermodular long-distance communications. However, negative connections between connector hubs are removed by traditional hard-thresholding approach in graph-theoretical research. Using soft-thresholding approach to reserve negative links, we explore time-varying features of connector hubs in intermodular communications during cognitive control of affective pictures. We develop a novel approach to sparse functional networks and construct negatively linking connector networks for positive, negative, and neutral pictures. We find that consisting of flexible hubs, the frontoparietal system dynamically top–down inhibits neural activities through negative connections from the salience subnetwork and visual processing area. Moreover, the shared connectors form functional backbones that dynamically reconfigure according to differently-valenced pictures in order to coordinate both stability and flexibility of cognitive connector networks. These results reveal the necessity of conserving negative links between intermodular communications in chronnectome research and deepen the understanding of how connector networks dynamically evolute during cognitive control of affective processing.

## Introduction

The ability to cognitive control of emotions is critical to behavioral flexibility and well-being (Ochsner et al., 2012; Marek et al., 2015). Although patterns of activation during cognitive control of emotions have been well-characterized, graph theory-based connectivity research has shown that the brain network composes of functionally separate subnetworks or modules (Buckner et al., 2009; Barret and Satpute, 2013; Sripada et al., 2014). Furthermore, functional connectome research has revealed that affective processing at least contains frontoparietal subnetwork (e.g., dorsolateral prefrontal cortex, inferior and superior parietal lobule), salience subnetwork (e.g., insula, middle, and posterior cingulate cortex), visual subnetwork (within the occipital lobe), and cerebellum (Wolf et al., 2009; Lindquist and Barrett, 2012; Oosterwijk et al., 2012). However, with the emergence of chronnectome (Calhoun et al., 2014), little is known about how affective information is dynamically represented through distributed large-scale brain networks.

During affective processing, cognitive control relies on not only short-distance intramodular processing but also long-distance intermodular communications (Mesulam, 1990; Wager et al., 2008; Barret and Satpute, 2013). As a matter of fact, whole-brain networks contain some important long-distance communications (e.g., long intra/inter-hemispheric connections) (Sepulcre et al., 2010; Hermundstad et al., 2013). In graph-theoretic research, hubs integrate and distribute information in powerful ways and are further classifies into provincial and connector hubs (Power et al., 2013; Van den Heuvel and Sporns, 2013). Different from provincial hubs that connect nodes within a module, connector hubs with high participant coefficients connect to multiple communities and have access to a variety of types of different information (Power et al., 2011). Particularly, the focal damage to a connector hub (e.g., a frontoparietal hub) caused large-scale long-distance functional deficits (Gratton et al., 2012). Therefore, focusing on the role of connector hubs is crucial for understanding of intermodular communication during cognitive control of affective pictures. However, static connectomics did not capture temporal structure of brain activity (Calhoun et al., 2014). Recently, more and more research has become interested in understanding network dynamics that support emotional or cognitive states (Fornito et al., 2012; Shine et al., 2016; Betzel et al., 2017), learning (Mantzaris et al., 2013), development (Gu et al., 2015), aging (Betzel et al., 2014), and disease progression (Raj et al., 2015). Time-vary features of functional network (e.g., connector hubs) are import for understanding how functional network dynamically evolute during information communication (Sizemore and Bassett, 2018), which further deepens our understanding the nature of temporal dynamics of brain activity (Vidaurre et al., 2018).

To obtain a sparse adjacent matrix in graph theoretic analysis, the traditional hard-thresholding approach eliminates weak and negative couplings between nodes because these negative links are often conceived as noise or minor (Rubinov and Sporns, 2010, 2011). However, not all negative connections are biologically meaningless (Chang and Glover, 2009; Fornito et al., 2013). For instance, during cognitive reappraisal of affective pictures, there exist strong anticorrelations between prefrontal cortex (PFC) (e.g., dorsomedial PFC or dorsolateral PFC) and salience/limbic areas (e.g., insula or amygdala) (Ochsner et al., 2002; Urry et al., 2006; Drabant et al., 2009). Undeniably, negative connections between modules are quite pervasive in functional network architectures and play an important role in behavior (Fox et al., 2005; De pisapia et al., 2012; Fornito et al., 2013), particularly in the task-evoked networks, although they were rarely mentioned. Simply removing these negative links lead to inaccurate representation of affective networks. The reservation of internetwork anti-correlations are critical for the precise representation of connector hubs in graph theoretical analysis because connector hubs are responsible for intermodular communication (Buckner et al., 2009; Van den Heuvel and Sporns, 2011). In contrast, the soft-thresholding approaches to mapping [−1,1] to [0, 1] not only avoid issues related to network fragmentation but also simply suppress rather than remove weaker connections (Schwarz and McGonigle, 2011). Here we aimed to use soft-thresholding approach to explore time-varying features of connector hubs in intermodular communication during affective information processing.

This study aimed to explore how connector networks dynamically evolute during cognitive control of affective processing. We collected fMRI data from 26 right-handed healthy undergraduates while they performed a cognitive task (silently counting backward) during the viewing of affective (positive, negative, and neutral) pictures. After preprocessing, time-series of 278 whole-brain parcels were extracted and subjected to a wavelet decomposition (Shen et al., 2013). Then, we estimated the dynamic correlation coefficients (DCCs) of all possible pairs of 278 parcels (Lindquist et al., 2014) and obtained 27 group-averaged DCC matrices. These DCC matrices were subsequently Fisher z-transformed and subjected to soft-thresholding. Next, we used the BCT toolbox^1^ to calculate graph theoretic features of these full-connected matrices. Finally, we constructed and characterized connector networks for affective pictures across 9 time points. Based on the previous study of dynamic networks (Fornito et al., 2012; Shine et al., 2016; Betzel et al., 2017; Sizemore and Bassett, 2018; Vidaurre et al., 2018), we expected that connector networks would exhibit valence-dependent dynamic connectivity patterns over times; in order to coordinate the stability and the flexibility of connector networks during dynamic affective processing, we expected that the shared connector would keep robust connections and form the functional backbone correspondent with structural backbone (Hagmann et al., 2008; Liang et al., 2013; Zhang et al., 2015).

## Materials and Methods

### Participants

The participants consisted of 26 right-hand undergraduates (14 females, 12 males; mean age 21.25 ± 2.34 years). The participants self-reported having normal or corrected-to-normal visual acuity and had no prior history of psychiatric or neurological disorders. Data from one male and one female were ruled out because of poor-quality data collection. All the participants provided the written informed consent before the experiment and were paid for their participation after the experiment. All experiments were performed in accordance with relevant guidelines and regulations. This study was approved by the Ethical Committee of School of Psychology at East China Normal University.

### Materials

Stimuli consisted of 81 pictures from the Chinese Affective Picture System (Bai et al., 2005), which is a collection of standardized photographic materials from the International Affective Picture System. The images were grouped into three conditions: 27 positive pictures (e.g., a smiling face), 27 negative pictures (e.g., a wreckage), and 27 neutral pictures (e.g., a houshold object). There exist significant valence differences among the three picture categories [*F*(2,78) = 90.25, *p* < 0.001; *M* ± *SD*: Positive = 7.35 ± 0.14; Neutral = 4.79 ± 0.07; Negative = 2.41 ± 0.12]. The positive and negative pictures significantly differ from the neutral ones [*F*(1,75) = 54.62, 59.21, *p* < 0.001] but do not significantly differ from each other [*F*(1,75) = 1.28, *p* > 0.05; *M* ± *SD*: Positive = 5.69 ± 0.39; Neutral = 4.45 ± 0.41; Negative = 5.58 ± 0.36]. We used E-prime1.1 software (Psychology Software Tools, Pittsburgh, PA, United States) to present these images and control their timing.

### Task

**FIGURE 1:**
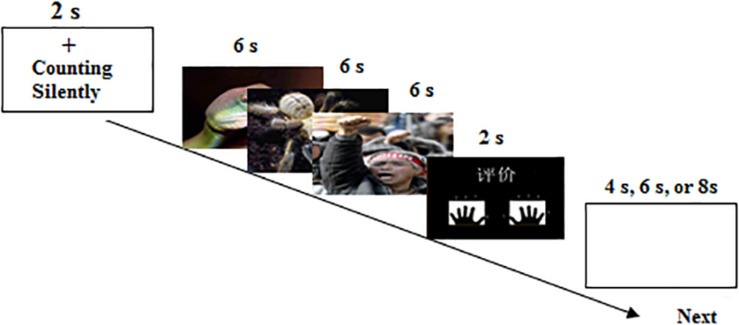
The illustration of the experimental procedure in the scanner.

On full trials (see Figure 1), a cue of the Chinese word “counting silently” under a fixation cross was first presented for 2 s. Then, three images with the same valence were continuously presented for 18 s, 6 s per picture. Once the cue emerged on the screen, the participants were required to count backward from 100 by three’s, i.e., 100, 97, 94, …, until all the pictures vanished. During counting silently, the participants always viewed the pictures. On the next screen the Chinese word “Assessment” appeared for 2 s, which prompted the participants to rate the arousal level of the pictures on a nine-point scale (1 = extremely weak to 9 = extremely strong) by pressing a button. Following the rating, a 4-, 6-, or 8-s jittered blank screen concluded the trial. The experiment contained 27 trials, 9 trials per picture type. The trial order was randomized and counterbalanced across subjects. After the fMRI experiment completed, the participants were asked to offline rate the arousal level using the similar paradigm with simply viewing 81 pictures outside the scanner. Before entering the scanner, the participants were familiar with the experimental procedure.

### fMRI Acquisition

Structural and functional MRI data were obtained with a 3-T Trio Tim Magnetic Resonance Imaging scanner (Siemens company) at East China Normal University. Foam padding was used to minimize head movement. Whole-brain functional data were acquired using a BOLD contrast-sensitive echo-planar T2^∗^-weighted imaging (EPI) sequence (32 axial slices, slice thickness = 5 mm; TR = 2000 ms, TE = 30 ms, flip angle = 90°, field of view = 240 mm × 240 mm, matrix size = 64 × 64, voxel size = 3.75 mm × 3.75 mm × 5 mm). The first two functional volumes were discarded to allow for equilibration effects. A T1-weighted anatomical image was obtained for each participants with 1 mm × 1 mm × 1 mm voxels (TR = 1900 ms, TE = 3.43 ms, flip angle = 7°).

### fMRI Analysis

The preprocessing of functional data was carried out using the SPM8 software package^2^. After functional data were corrected for slice timing and head motion, the T1-weighted images were coregistered to the mean EPI image. The coregistered data were spatially normalized into the standard MNI space and resampled to 3 mm isotropic voxels. Images were high-filtered with a cut-off of 128 s to remove low-frequency signal drift.

### Network Construction

The whole brain was parcellated into a set of subunits with 2 mm resolution that correspond to the 278 cortical and subcortical structures (Shen et al., 2013). These subunits were obtained using resting-state fMRI and have been shown to be highly functionally homogeneous and spatially coherent. For each individual fMRI dataset, regional mean BOLD time-series of each subunit were extracted using the marsbar toolbox^3^ separately for the positive, negative, and neutral picture conditions. These regional time-series were then subjected to a wavelet decomposition to reconstruct wavelet coefficients in the 0.03–0.07 Hz range using the wmtsa toolbox^4^. Unlike the traditional assumption that the functional connectivity between time series from distinct brain regions is constant across time, the DCC provides a model-based approach toward estimating dynamic correlations and achieves the best overall balance between sensitivity and specificity in detecting dynamic changes in correlations (Lindquist et al., 2014). Therefore, we used the DCC toolbox (Lindquist et al., 2014) to estimate DCCs of these wavelet coefficients time-series between all possible pairs of 278 brain subunits and averaged the 278 × 278 functional connectivity matrices over all trials and all participants. The resulting 27 group-averaged DCC matrices (9 TRs × 3 picture conditions) were Fisher z-transformed.

### Graph-Theoretic Analysis

To conserve negative and weak edges, we used the soft-thresholding approach to map a correlation coefficients r_*ij*__∈_[−1, 1] to a weight w_*ij*__∈_[0, 1]. These mappings have recently been evaluated in a study of the modular organization of the brain from functional connectivity data (Mumford et al., 2010). This approach can replace the hard thresholding with a continuous mapping of all correlation values to edge weights, suppressing rather than removing weaker connections and avoiding issues related to network fragmentation. Here, a soft-thresholding adjacency function was defined by a power function (Schwarz and McGonigle, 2011), w_*ij*_ = [(r_*ij*_ + 1)/2]^12^. We used the BCT toolbox to compute the following four graph-theoretic characteristics of parcel-wise whole-brain networks. The first analysis used Louvain algorithm to identify community structures of full-connection networks of 278 nodes at each time point. Then, we computed the participation coefficients to examine the role of hubs in interconnecting distinct modules. We also calculated nodal betweenness centrality and edge betweenness centrality. In this study, the hubs with participation coefficient > 0.5 and both node betweenness centrality and edge betweenness centrality ≠ 0 at each time point were classified into connector hubs. These connector hubs describe the level of intermodule connectivity and facilitate the integration of multiple types of information during intermodular communication. To sparse the full-connected DCC matrices, we binarized the matrices of edge betweenness centrality and then dot-multiplied them by the correspondent DCC matrices. That is, we retained the connectivity values between two connectors in the DDC matrices only if their edge betweenness centrality value was not equal to zero. Thus, we obtained the connector networks.

## Results

### Behavioral Data

**FIGURE 2:**
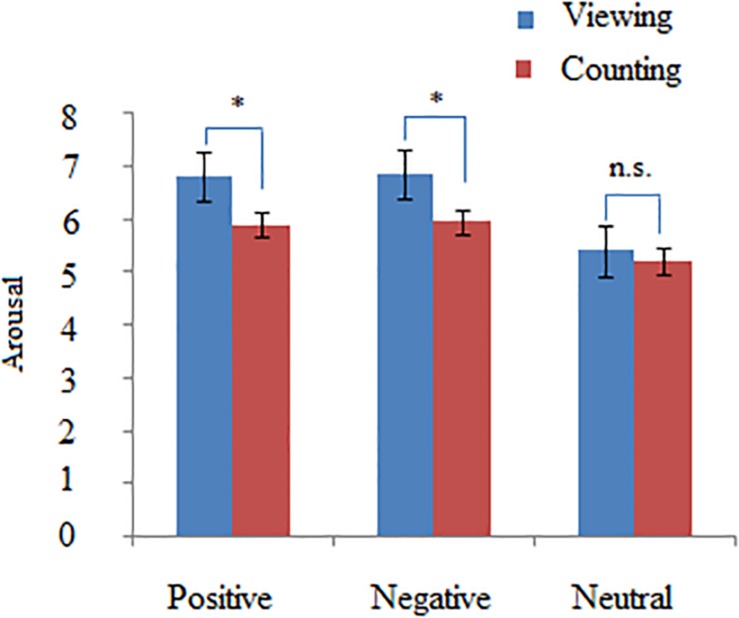
The statistical results of arousal rating in the Counting vs. Viewing condition. The ANOVA analysis with picture types (positive, negative, and neutral) and task (Counting and Viewing) as within-subject factors indicated that compared with Viewing, Counting task significantly decreased the arousal levels of positive and negative pictures but not those of neutral pictures. ^∗^*p* < 0.05.

In order to verify if silently counting validly reduce the emotional responses evoked by affective picture, a two-way repeated measure ANOVA with valence (positive, neutral, and negative) × task (silently counting and simple viewing) revealed a significant main effect of valence on arousal rating [*F*(2,46) = 10.25, ${\text{η}}_{\text{p}}^{2}$ = 0.39, *p* < 0.001] and a significant interaction between valence and task [*F*(2,46) = 9.43, ${\text{η}}_{\text{p}}^{2}$ = 0.32, *p* < 0.001]. *Post hoc* tests indicated that the counting task produced a lower arousal rating for positive and negative pictures relative to simple viewing (*p* < 0.01), but not for neutral pictures (*p* > 0.05) (see Figure 2). These results suggest that participants were involved in the cognitive control of affective picture processing within the scanner.

### Identification of Connector Hubs

We calculated several graph-theoretic features to examine the role of hubs in interconnecting distinct modules. Participant coefficient is a measure of diversity of intermodular connections of individual nodes (Van den Heuvel and Sporns, 2011). Nodes with high participant coefficient display high correlation with multiple modules, which facilitates integration of multiple types of information (Warren et al., 2014). Nodal betweenness centrality measures the influence of a node on information flow between additional nodes in the network (Hagmann et al., 2008). Similarly, edge betweenness centrality measures the influence of an edge on information flow (Brandes, 2001). At all 9 time points, nodes with participation coefficient > 0.5 and both nodal betweenness centrality and edge betweenness centrality ≠ 0 were classified as connector hubs. We retained the connector hubs that have at least a connection with other connector hubs in different communities. Finally, we binarized edge betweenness centrality matrices and dot-multiplied them by the correspondent DCC matrices. Thus, we sparsed the full-connected DCC matrices into connector networks (Supplementary Figure S1).

**FIGURE 3:**
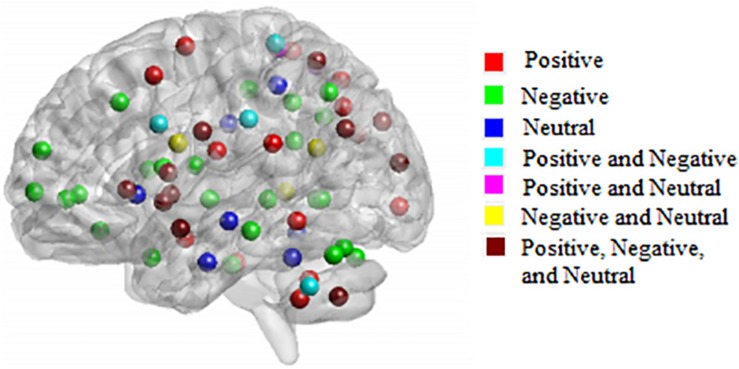
Neuroanatomical location of 65 connector hubs for three picture types. Red, for positive pictures; green, for negative pictures; blue, for neutral pictures; cyan, intersection between positive and negative pictures; fuchsia, intersection between positive and neutral pictures; yellow, negative and neutral pictures; maroon, intersection among positive, negative, neutral pictures.

Figure 3 shows 65 connector hubs for positive, negative, and neutral pictures for their coordinates. The connector networks contained 31 nodes for positive pictures (see Table 1), mainly including the frontoparietal nodes (e.g., inferior and middle frontal gyrus, inferior and superior parietal lobule), the salience nodes (e.g., insula, putamen, caudate, amygdala), and the visual nodes (e.g., cuneus, middle and superior occipital gyrus). For negative pictures, the connector network contained 42 nodes (see Table 2) and their mayjority were located in the frontoparietal area (e.g., inferior and superior frontal gyrus, inferior and superior parietal lobe, precentral gyrus, angular gyrus), the salience system (e.g., insula, putamen, cingulate cortex, hippocampus), and the cerebellum. For neutral pictures, the connector network included 23 nodes (see Table 3) and were mainly found in the frontoparietal area (inferior frontal gyrus, superior parietal lobe, post-central gyrus), the salience system (insula, putamen, posterior cingulate cortex), and the visual area (cuneus, superior occipital gyrus). Moreover, all the connector networks were always composed of seven modules (see Supplementary Figure S2) and each module possessed more than one connectors.

**TABLE 1:** Connector hubs for positive pictures.

**Node**	**Subunit**	**BA**	**AAL**	**Talairach**		
				
				**X**	**Y**	**Z**
1	115	−	Pons_R	7	−23	−26
2	133	R.BA53.1	Amygdala_R	25	−2	−16
3	149	L.BA49.1	Putamen_L	−23	4	2
4	154	L.BA4.1	Pretcentral_L	−57	−9	28
5	159	−	Culmen_L	−44	−53	−36
6	165	L.BA18.8	Cuneus_L	−4	−90	15
7	169	L.BA45.2	Insula_L	−32	22	4
8	174	−	Cerebellum_8_L	−20	−65	−40
9	177	L.BA6.4	Inferior Frontal Gyrus	−41	9	31
10	190	L.BA7.6	Occipital_Sup_L	−14	−68	29
11	195	L.BA40.1	Inferior Parietal Lobule	−57	−28	33
12	201	L.BA37.1	Temporal_Mid_L	−57	−48	−9
13	203	L.BA6.6	Middle Frontal Gyrus	−24	11	51
14	211	−	Cerebellar Tonsil	−27	−49	−41
15	216	L.BA13.2	Insula_L	−36	7	−1
16	223	L.BA19.9	Cuneus_L	−10	−83	32
17	240	L.BA7.4	Parietal_Sup_L	−21	−56	57
18	244	L.BA38.1	Temporal_Sup_L	−46	0	−12
19	245	L.BA40.3	Inferior Parietal Lobule	−55	−38	23
20	249	L.BA7.9	Precuneus_L	−3	−67	38
21	255	L.BA7.7	Precuneus_L	−12	−39	64
22	260	L.BA48.1	Caudate_L	−14	−15	20
23	269	L.BA44.2	Insula_L	−35	5	11
24	277	L.BA6.7	Supp_Motor_Area_L	−14	−2	63
25	29	R.BA7.6	Superior Parietal Lobule	24	−46	61
26	39	R.BA19.5	Occipital_Mid_R	43	−73	27
27	45	R.BA7.3	Paracentral_Lobule_R	7	−40	62
28	60	R.BA7.5	Superior Parietal Lobule	23	−66	49
29	66	R.BA20.3	Cerebelum_Crus1_R	46	−53	−33
30	80	R.BA7.4	Parietal_Sup_R	21	−56	55
31	96	R.BA18.5	Occipital_Inf_R	23	−90	−3

*The first column denotes the nodal number in the Pajek graphs. The second column represents the subunit codes of whole-brain parcellation by Shen et al. (2013). BA, Broadmann’s area; AAL, automated anatomical labeling.*

**TABLE 2:** Connector hubs for negative pictures.

**Node**	**Subunit**	**BA**	**AAL**	**Talairach**		
				
				**X**	**Y**	**Z**
1	111	R.BA44.2	Frontal_Inf_Oper_R	52	13	12
2	117	R.BA19.1	Lingual_R	14	−58	0
3	123	R.BA38.2	Temporal_Pole_Sup_R	37	12	−24
4	134	R.BA47.2	Frontal_Inf_Orb_R	26	33	−13
5	136	R.BA36.2	Precuneus_R	15	−43	3
6	149	L.BA49.1	Putamen_L	−23	4	2
7	154	L.BA4.1	Pretcentral_L	−57	−9	28
8	158	−	Pons	−4	−21	−27
9	159	−	Culmen_L	−44	−53	−36
‘10	162	L.BA37.3	Cerebellum_Crus2_L	−46	−63	−23
11	165	L.BA18.8	Cuneus_L	−4	−90	15
12	169	L.BA45.2	Insula_L	−32	22	4
13	171	L.BA23.1	Posterior Cingulate	−8	−56	21
14	174	−	Cerebelum_8_L	−20	−65	−40
15	177	L.BA6.4	Inferior Frontal Gyrus	−41	9	31
16	190	L.BA7.6	Occipital_Sup_L	−14	−68	29
17	194	L.BA39.5	Angular_L	−39	−61	44
18	195	L.BA40.1	Inferior Parietal Lobule	−57	−28	33
19	200	L.BA10.4	Frontal_Sup_L (aal)	−16	60	3
20	202	L.BA19.6	Cerebellum_6_L	−26	−67	−20
21	206	L.BA6.1	Inferior Frontal Gyrus	−53	1	23
22	207	L.BA13.3	Insula_L	−35	−12	0
23	216	L.BA13.2	Insula_L	−36	7	−1
24	223	L.BA19.9	Cuneus_L	−10	−83	32
25	225	L.BA10.7	Cingulum_Ant_L	−7	47	0
26	238	L.BA21.3	Temporal_Mid_L	−56	−29	−13
27	240	L.BA7.4	Parietal_Sup_L	−21	−56	57
28	244	L.BA38.1	Temporal_Sup_L	−46	0	−12
29	24	R.BA39.2	Temporal_Sup_R	48	−59	34
30	251	L.BA8.2	Frontal_Sup_L	−21	25	40
31	255	L.BA7.7	Precuneus_L	−12	−39	64
32	267	L.BA10.6	Frontal_Sup_Medial_L	−10	57	20
33	269	L.BA44.2	Insula_L	−35	5	11
34	273	L.BA48.2	Caudate_L	−12	7	13
35	35	R.BA37.8	Temporal_Mid_R	54	−56	0
36	42	R.BA54.2	Hippocampus_R	30	−36	0
37	55	R.BA31.2	Cingulum_Mid_R	9	−28	45
38	75	R.BA39.4	Inferior Parietal Lobule	58	−47	24
39	81	R.BA10.1	Cingulum_Ant_R	8	42	2
40	86	R.BA37.7	Cerebellum_Crus1_R	47	−72	−24
41	93	R.BA13.3	Insula_R	40	−6	14
42	94	R.BA40.3	Parietal_Inf_R	51	−46	40

**TABLE 3:** Connector hubs for neutral pictures.

**Node**	**Subunit**	**BA**	**AAL**	**Talairach**		
				
				**X**	**Y**	**Z**
1	136	R.BA36.2	Precuneus_R	15	−43	3
2	149	L.BA49.1	Putamen_L	−23	4	2
3	154	L.BA4.1	Pretcentral_L	−57	−9	28
4	165	L.BA18.8	Cuneus_L	−4	−90	15
5	169	L.BA45.2	Insula_L	−32	22	4
6	171	L.BA23.1	Posterior Cingulate	−8	−56	21
7	174	−	Cerebellum_8_L	−20	−65	−40
8	190	L.BA7.6	Occipital_Sup_L	−14	−68	29
9	198	L.BA20.1	Temporal_Inf_L	−49	−11	−26
10	206	L.BA6.1	Inferior Frontal Gyrus	−53	1	23
11	216	L.BA13.2	Insula_L	−36	7	−1
12	223	L.BA19.9	Cuneus_L	−10	−83	32
13	224	L.BA31.1	Precuneus_L	−8	−40	47
14	240	L.BA7.4	Parietal_Sup_L	−21	−56	57
15	244	L.BA38.1	Temporal_Sup_L	−46	0	−12
16	252	L.BA37.6	Fusiform_L	−32	−46	−24
17	263	L.BA48.3	Caudate_L	−11	18	2
18	266	L.BA54.1	−	−28	−20	−9
19	269	L.BA44.2	Insula_L	−35	5	11
20	45	R.BA7.3	Paracentral_Lobule_R	7	−40	62
21	5	R.BA40.4	Postcentral_R	57	−20	31
22	80	R.BA7.4	Parietal_Sup_R	21	−56	55
23	90	R.BA37.9	Temporal_Inf_R	54	−48	−14

### Time-Varying Connector Networks in Affective Processing

**FIGURE 4:**
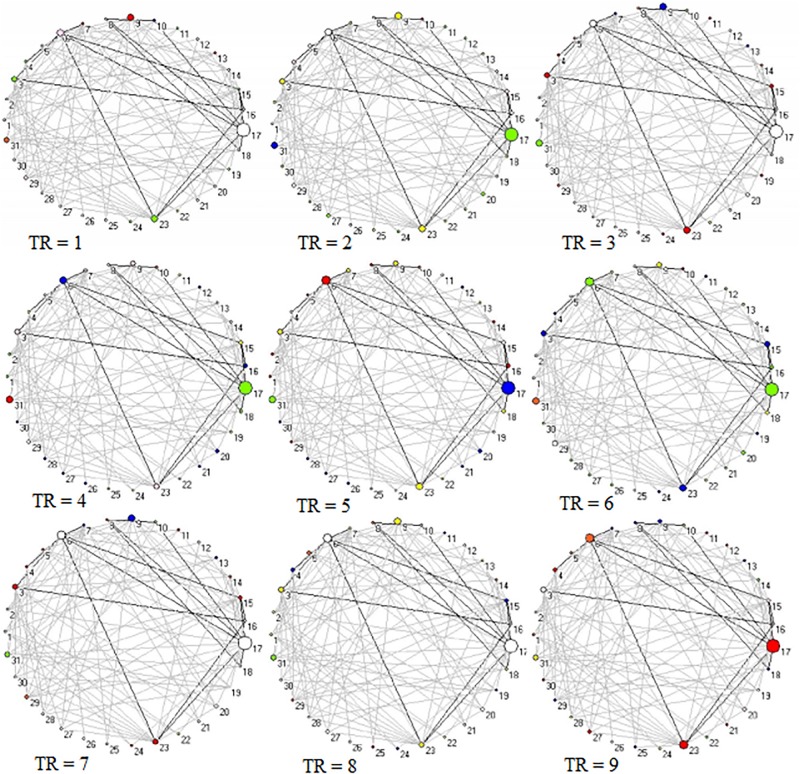
Two-dimension depiction of connector networks for positive pictures at 9 time points. The nodal color denotes affiliative community; the nodal size represents the magnitude of nodal betweenness centrality; the edge depicts binarized edge betweenness centrality and all the edges are negative. The solid lines represent the functional backbone. The vertex number was consistent with Table 1. The graphs were plotted by the Pajek package.

**FIGURE 5:**
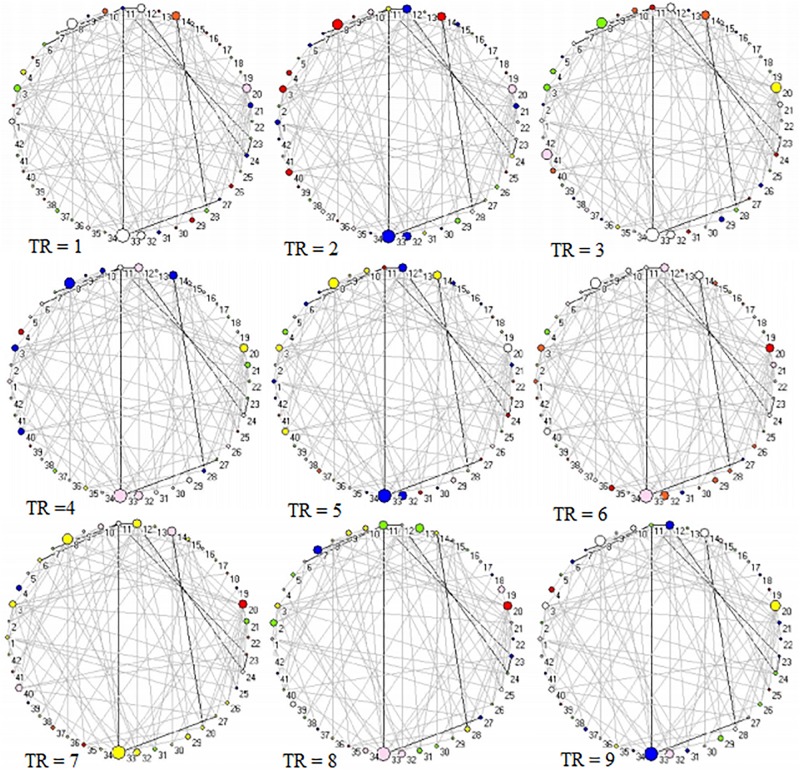
Two-dimension depiction of connector networks for negative pictures at 9 time points. The vertex number was consistent with Table 2. The others were the same as Figure 3.

**FIGURE 6:**
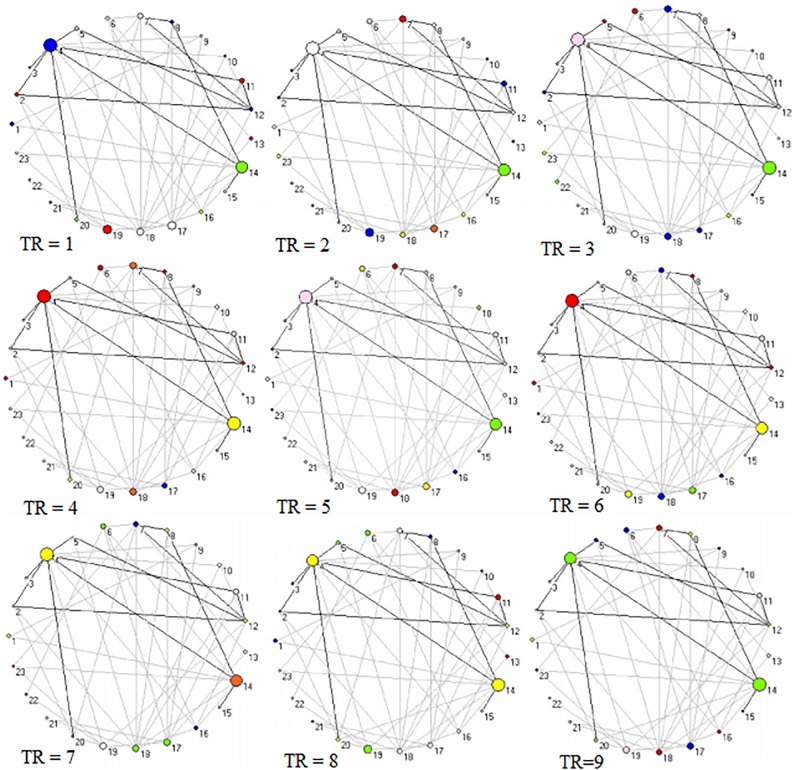
Two-dimension depiction of connector networks for neutral pictures at 9 time points. The nodal number was consistent with Table 3. The others were the same as Figure 3.

**FIGURE 7:**
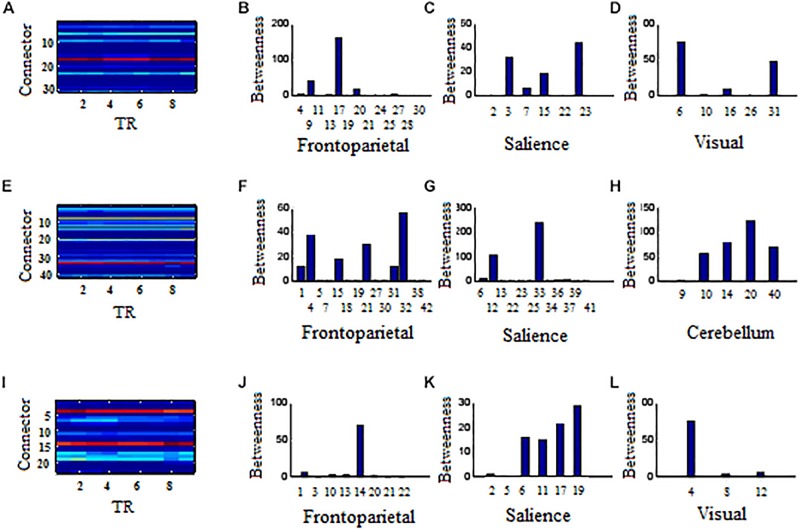
Affiliative community and nodal betweenness centrality of connector networks. **(A,E,I)** Affiliative community of connector network for positive, negative, and neutral pictures, respectively. **(B–D)** Bar graph of nodal betweenness centrality for positive picutres. **(F–H)** Bar graph of nodal betweenness centrality for negative pictures. **(J–L)** Bar graph of nodal betweenness centrality for neutral pictures. The node number was consistent with Tables 1–3, respectively for positive, negative, and neutral pictures. Bar graphs of nodal betweenness centrality at TR = 5 were used for example.

Across 9 time points (from TR = 1 to 9), the connector networks displayed different dynamic connectivity patterns when participants performed cognitive processing of positive, negative, and neutral pictures (see Figures 4–6, respectively). The DCC matrices of connector hubs exhibited time-varying patterns (see Supplementary Video S1 for positive, Supplementary Video S2 for negative, and Supplementary Video S3 for neutral online). However, regardless of picture valences, the connection values between connectors were always negative when the connectors belongs to different communities. Their affiliative communities also reconfigured dynamically over times (Figures 7A,E,I).

**FIGURE 8:**
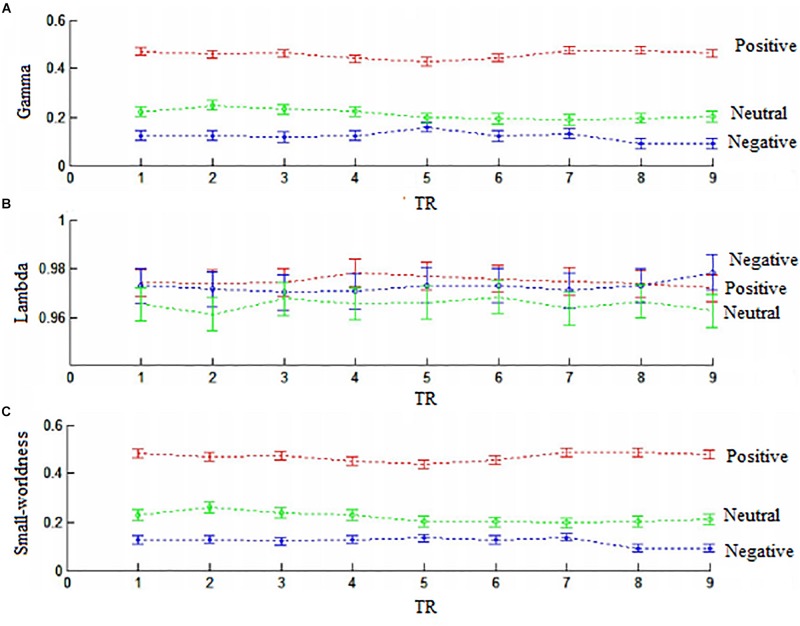
The samll-worldness of connector networks. **(A)** Gamma values (standardized cluster coefficients). **(B)** Lambda (standardized characteristic path length). **(C)** Small-worldness. Small-worldness = gamma/lambda. Positive, red color; negative, green clolor; neutral, blue color.

Next, we used the Gretna software^5^ to calculate the small-worldness value of connector networks. Small-worldness is a property of a network with high clustering but low characteristic path length (Yan et al., 2011). A real network is considered small-world if the ratio of cluster coefficient to characteristic path length is greater than one relative to random networks. Previous research has demonstrated that the small-world feature are quite universal in structural and functional networks (Varoquaux and Craddock, 2013). However, small-worldness in this study was less than one across nine time points (all *ps* < 0.01) because standardized cluster coefficients (gamma value) were significantly less than one (all *ps* < 0.01) but standardized characteristic path length (lambda value) were almost close to one. Thus, small-worldness did not emerge in connector networks (Figure 8).

#### Positive Pictures

In the frontoparietal system, the left superior parietal lobule (Node 17, BA7.4) had larger betweenness centrality value than other 12 connector hubs (*p* < 0.001, Figure 7B). In the salience system, betweenness centrality value of the left insula (Node 23, BA44.2) was larger than other five salience hubs (*p* < 0.01, Figure 7C). In the visual system, the left cuneus (Node 6, BA18.8) exhibited larger betweenness centrality value than other four hubs (*p* < 0.01, Figure 7D).

#### Negative Pictures

In the frontoparietal system, the superior medial frontal gyrus (Node 32, BA10.6) in the left hemisphere had larger betweenness centrality value than other 13 connector hubs (*p* < 0.001, Figure 7F). Of 13 hubs in the salience system, the left insula (Node 33, BA44.2) had the largest betweenness centrality value (*p* < 0.001, Figure 7G). The cerebellum had five connector hubs that involve negative processing, of which Node 20 exhibited the largest betweenness centrality value (*p* < 0.01, Figure 7H).

#### Neutral Pictures

In the frontoparietal system, the betweenness centrality value of the left superior parietal lobe (Node 14, BA7.4) was larger than other seven connector hubs (*p* < 0.001, Figure 7J). The left insula (Node 19, BA44.2) exhibited the largest betweenness centrality value in the salience sytem (*p* < 0.01, Figure 7K). In the visual system, the left cuneus had larger betweenness centrality value than other two hubs (*p* < 0.001, Figure 7L).

### Shared Hubs in the Functional Backbone

**FIGURE 9:**
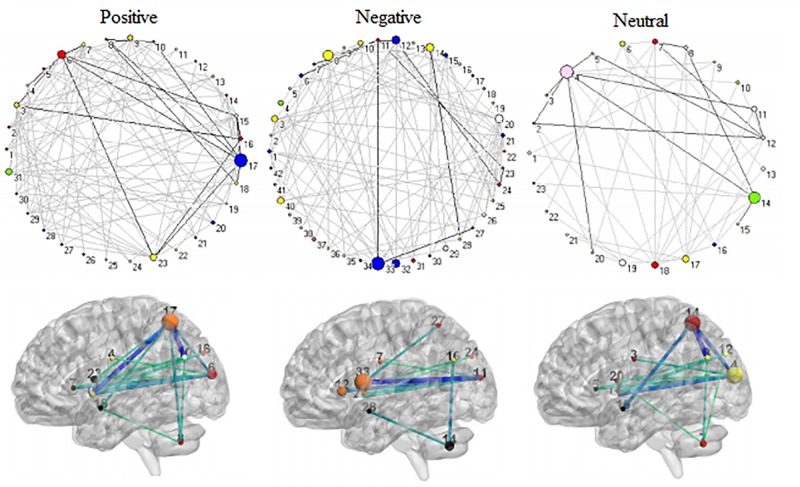
The shared 11 connector hubs of the functional backbone for positive, negative, and neutral pictures at TR = 5.

The connector networks for positive, negative, and neutral pictures always shared 11 hubs in the left hemisphere (Figure 9). They were involved in affective processing (e.g., insula, putamen, and cerebellum), visual processing (e.g., cuneus and superior occipital gyrus), and cognitive processing (e.g., superior parietal lobule, precentral gyrus, and superior temporal gyrus). After binarizing edge betweenness centrality matrices composed of 11 nodes, these matrices always kept invariant functional connectivity patterns on the same picture condition at all 9 time points (solid lines in Figures 4–6). Moreover, the shared 11 connectors always kept relatively robust during full trials. These shared connectors kept largely correspondent to structural cores with high metabolic activity in the structural backbone. Somewhat similar to a structural backbone in structural connectivity networks (Hagmann et al., 2008), the shared connector networks were tentatively called functional backbone (see secion “Discussion”), which is necessary for coordinating both the stability and the flexibility of cognitive control networks during dynamic affective processing.

## Discussion

The current study used graph theory approach to explore how connector hubs dynamically evolute during cognitive control of affective pictures. We used soft-thresholding to reserve negative connections (Schwarz and McGonigle, 2011). Results revealed that connector networks consisted of cross-modular negative connectivities, which is impossible to be unveiled by hard-thresholding. This might be because we were the first time to use edge betweenness centrality to sparse connector networks, different from traditional hard-thresholding that just removes weak links (Rubinov and Sporns, 2010, 2011). Edge betweenness centrality is the fraction of all shortest paths in the network that contain a given edge and reflects an edge’ centrality of in a network but not link strength (Brandes, 2001). Thus, negative links between connector hubs is not noise or spurious connections but take an important role in intermodular long-distance communications. Overall, this study not only provides a novel method to sparse functional networks but also reveals that there exist negatively linking connector networks during intermodular integration of affective information.

Connector networks exhibited valence-related dynamic connectivity patterns over times, e.g., dynamic community affiliations and time-varying connections with other parts of whole-brain networks. These dynamic changes facilitate intermodular communications of affective information. However, connector networks did not exhibit small-worldness because of low cluster coefficient but high characteristic path length, regardless of picture valences. This implies that these connector networks contain less direct links but more indirect links. For example, the frontoparietal subnetwork does not receive direct afferents from the visual system, but executes top–down control indirectly via the ventral attention/salience network, which contains the anterior insula (Seeley et al., 2007; Vossel et al., 2012; Palaniyappan et al., 2013). This study requested participants to perform the silent-counting task while they viewed pictures on the screen. Participants needed to inhibit visually affective information when pictures appeared on the screen. Behavioral results indicated that the counting task significantly decreased arousal rating for positive and negative pictures but not neutral pictures compared with offline viewing. Consistent with behavioral findings, the frontoparietal system (e.g., superior frontal gyrus and superior parietal lobe) through negative connections dynamically top–down inhibited the neural activities from salience subnetwork (e.g., insula) and visual area (e.g., cuneus).

Affective picture processing also exhibited different nodal betweenness patterns in connector networks. The present study found that the left superior parietal lobe (BA7.4) in the frontoparietal system exhibited larger nodal betweenness for positive and neutral pictures, although connector networks contained less hubs in the neutral picture condition than in the positive picture condition. In contrast, connector networks of negative pictures included larger nodal betweenness in the superior medial frontal gyrus (BA10.6). These results were consistent with the frontoparietal control system consisting of flexible hubs that integrate information from the external environment (e.g., visual stimuli) with stored internal representations (e.g., silently counting) and regulate distributed systems (e.g., visual, salience) according to affective valences (Vincent et al., 2008; Woolgar et al., 2015). Moreover, the left insula (BA44.2) had larger nodal betweenness centrality in the salience system of all connector networks. This might be attributed to that the insula regulates the interaction between the salience of the selective attention created to achieve a task and the salience of emotional arousal and has an important role in the experience of emotions (Taylor et al., 2009; Quarto et al., 2016).

In particular, five cerebellar hubs participated in network processing of negative pictures while fewer cerebellar connector hubs for positive and neutral pictures. As being famous for sensorimotor control (Bastian, 2011), the human cerebellum has connections with other brain areas involved in affective regulation, including the insula, amygdala, hypothalamus, as well as the neocortex (Baumann and Mattingley, 2012). Patients with cerebellar diseases had significant higher depressive scores relative to healthy comparison groups (Wolf et al., 2009). Neuroimaging studies revealed that the cerebellum was involved in control processing of aversive stimuli and other basic emotions (Moulton et al., 2011). However, cerebellar hubs were ignored by several cortical parcellations (e.g., Power et al., 2011; Yeo et al., 2011; Gordon et al., 2014). Future studies should follow in interest the centrality of cerebellar hubs in whole-brain network processing of affective information.

The shared 11 connectors kept robust connections and formed the functional backbone. The functional backbone mainly consisted of connector hubs with high metabolic activity and might be constrained by cortical structural cores (Hagmann et al., 2008). Different from structural connections, functional connectivities reflect statistical dependence between nodes and thus functional hubs have unnecessarily direct structural connection with other hubs (Sporns, 2014). Under the disturbing of active tasks, metabolic energy is necessarily redistributed more to task-specific hubs to complete tasks (Liang et al., 2013; Zhang et al., 2015). The present functional backbone exhibited different connectivity patterns among positive, negative, and neutral pictures, which reconfigured during cognitive control of differently-valenced affective processing.

From a perspective of temporal dynamics, the existence of the functional backbone helps not only maintain network integrity (or task set) across full trials but also flexibly integrate different types of information from different modules during cognitive processing. Therefore, the functional backbone plays a critical role in coordinating both the stability and the flexibility of cognitive control networks during dynamic processing of affective information. In particular, the connector lesions influence processing in multiple systems and produces impairment across several cognitive domains (Gratton et al., 2012). Therefore, the functional backbone concept has important implications for the understanding the relationship between disruption of whole-brain network architecture and functional deficits in neurological or psychiatric disorders. Future work will need to further examine the importance of functional backbone of connector networks in clinical research with other experimental paradigms.

The limitations in this study must be mentioned as follows. First, the sample size is small and future studies should use larger sample to confirm the results. Second, dynamic networks are a very active and fast field. Recently, some novel approaches on temporal dynamic of brain networks have been put forward (Sizemore and Bassett, 2018; Vidaurre et al., 2018). Future studies should use these new approaches to further investigate how connector networks dynamically evolute and verify the importance of the functional backbone. Third, since the hard- and soft-thresholding have different ideas about how to deal with weak and negative connections, future studies might use the simulating methods to completely compare the soft-thresholding approach with the traditional approach so as to further clarify their appropriateness.

In the summary, the present findings lay stress on the necessity of conserving negative connections between multiple modules in chronnectome research, and provide evidence for the existence of functional backbone during dynamic evolution of the connector networks. These results deepen the understanding of the relationship between structural and functional connectivity during dynamic processing of affective information.

## Data Availability Statement

The raw data supporting the conclusions of this manuscript will be made available by the authors, without undue reservation, to any qualified researcher.

## Ethics Statement

All subjects gave written informed consent in accordance with the Declaration of Helsinki. The protocol was approved by the Ethical Committee of School of Psychology at East China Normal University.

## Author Contributions

QL, GR, and WZ designed and performed the study and analyzed the data. WZ wrote the manuscript. FT, HL, GR, and JC reviewed and modified the manuscript.

## Conflict of Interest

The authors declare that the research was conducted in the absence of any commercial or financial relationships that could be construed as a potential conflict of interest.

## Footnotes

1 http://www.brain-connectivity-toolbox.net

2 https://www.fil.ion.ucl.ac.uk/spm/software/spm8/

3 http://marsbar.sourceforge.net/

4 http://cran.r-project.org/web/packages/wmtsa/index.html

5 www.nitrc.org/frs/download.php/5534/gretna.zip
