# Supplementary material for: Negatively Linking Connector Networks in Cognitive Control of Affective Pictures
Source: Front Neurosci. 2019 Oct 25;13:1069. doi: 10.3389/fnins.2019.01069 (PMC6823191; doi:10.3389/fnins.2019.01069)
Supplement: Supplementary file 4 [file Data_Sheet_1.doc]

**Supporting Information**

**S1 Fig. Sparsed connectivity matrices of connector networks for positive, negative, and neutral pictures.** (TIF)

**S2 Fig. Affiliative community of whole brain nodes for positive (a), negative (b), and neutral (c) pictures.** (TIF)

**S1 Video. Time-varying DCC matrices of connector networks for positive pictures at TR =1 to 9.** The DCC matrices were fully connected and belonged to [-1, 1]. The nodal order was consistent with Table 1, 2, and 3. The cool color represents negative values and the warm color represents positive values. (AVI)

**S2 Video. Time-varying DCC matrices of connector networks for negative pictures at TR =1 to 9.** (AVI)

**S3 Video. Time-varying DCC matrices of connector networks for neutral pictures at TR =1 to 9.** (AVI)
