# Supplementary figures and images for: Negatively Linking Connector Networks in Cognitive Control of Affective Pictures
Source: Front Neurosci. 2019 Oct 25;13:1069. doi: 10.3389/fnins.2019.01069 (PMC6823191; doi:10.3389/fnins.2019.01069)

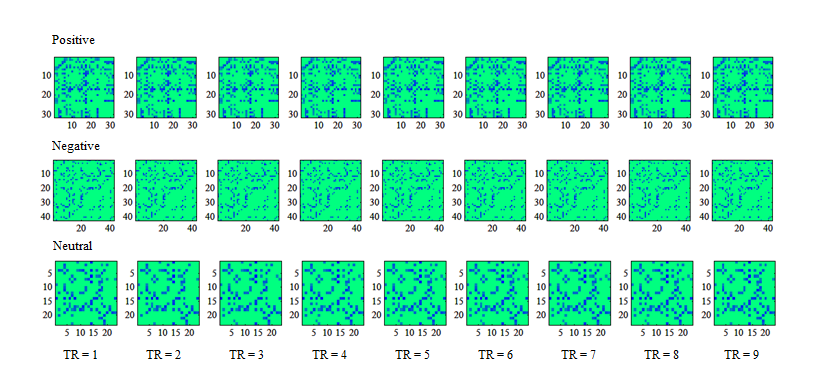

Supplement: Supplementary file 5 [file Image_1.TIF]

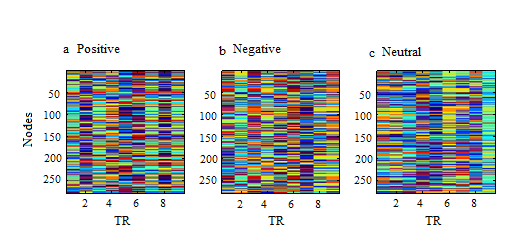

Supplement: Supplementary file 6 [file Image_2.TIF]
